# Topical Nitric Acid Burns: Initial Assessment and Management

**DOI:** 10.1155/2023/9995933

**Published:** 2023-07-24

**Authors:** Asmaa Laanaya, Mehdi Ami Ali, Amal Miqdadi, Mustapha Noussair, Mourad Nafaa, Lahcen Belyamani

**Affiliations:** ^1^Emergency Department, Cheikh Khalifa International University Hospital, Faculty of Medicine, Mohammed VI University of Health and Sciences, Casablanca, Morocco; ^2^Emergency Department, Mohamed V Military Hospital, Mohammed V University, Rabat, Morocco

## Abstract

Nitric acid (NA) is corrosive. On contact with the skin, liquid splashes with nitric acid and may produce severe burns. These burns usually take on a characteristic yellowish hue. We report the case of a 54-year-old man presenting a nitric acid burn with a pathognomonic skin lesion and perfect healing. NA is a chemical that is important in industry, and it is a very strong acid that is used for engraving, metal refining, electroplating, and fertilizer manufacturing. Skin contact with NA leads to severe burns. The pathophysiology depends on the type of concentration, the strength, quality, and duration of contact, and the penetration power of the acids concerned. The early and abundant irrigation with water or sterile isotonic saline solution, the use of panthenol-containing creams and covering with silver sulphadiazine dressing, carefully monitoring wounds, keeping wounds clean and moist, and preventing and managing secondary infection allow the healing.

## 1. Introduction

Chemical burn injuries, including nitric acid injuries, are rarely encountered in routine daily practice, but they cause a particular type of lesion in which disability is high and aesthetic sequelae are very important [1]. Nitric acid (NA) is known to be a highly corrosive liquid, which on skin contact leads to severe burns, and its vapours can cause severe acid burns to the eyes, respiratory tract, and lungs [1]. A few publications addressing this topic can be found in the medical database with only a few images of these injuries with no guidelines about the management of the disease [2]. We report the case of a nitric acid burn with a pathognomonic skin lesion and the follow-up on his process of healing. The purpose is to make the emergency diagnosis quicker, to raise awareness about the urge for monitoring, and to have guidelines about the management of this disease.

## 2. Case Report

**Figure 1:**
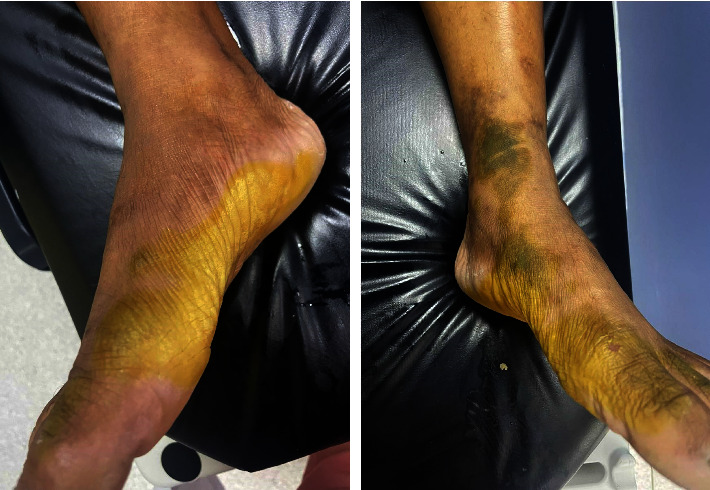
The characteristic nitric acid burn wound on the inner edges of the feet.

A 54-year-old man, with no pathological history, who had received nitric acid burns on the inner edges of the feet and internal malleolus, was admitted to the emergency two hours after the accident (work accident) with the characteristic NA burn wound, and the degree of burn injury was between second and third degree (Figure 1).

After abundant washing of lesions with an isotonic saline solution (the antidote was not available), the wounds were covered with a sulphadiazine cream and an antiseptic moist bandage.

**Figure 2:**
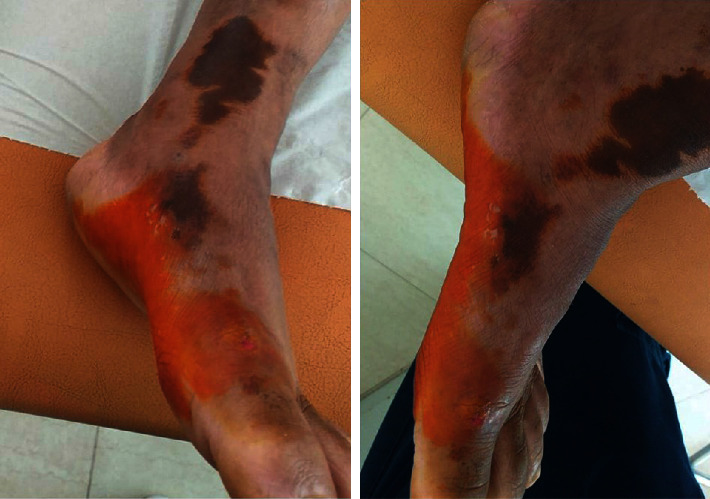
Nitric acid injury color on the inner edges of his feet changed to a yellow stain 24 h after the injury.

24 hours later, the skin color changed to a yellow stain (Figure 2).

**Figure 3:**
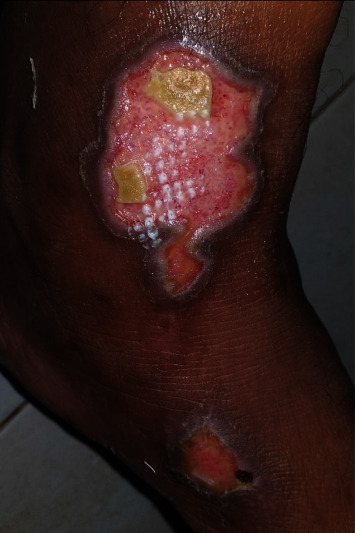
The appearance of wounds from healing buds all around was observed one month after the injury.

**Figure 4:**
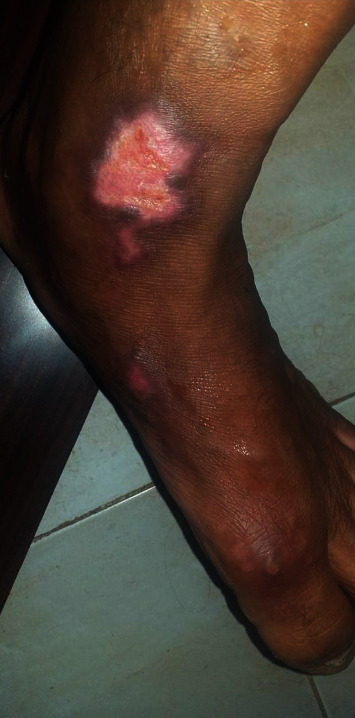
The skin regeneration is observed two months after the injury.

The evolution after one month was marked by the appearance of wounds from healing buds all around with no sign of infection (Figure 3). The same treatment was continued for one more month, following daily dressing changes, and all wounds healed adequately (Figure 4).

## 3. Discussion

Nitric acid is a chemical that is important in the industry, it is a very strong acid and a powerful oxidizing agent with the ability to nitrate organic materials, and it is used for engraving, metal refining, electroplating, and fertilizer manufacturing [1, 2].

The diagnostic key here is the yellowing of the skin at the injury site. This yellowish hue is the characteristic of chemical injury secondary to nitric acid exposure [3].

The physiopathology depends on several factors, including penetration power, the type of concentration, quality, strength, and duration of contact with the acids concerned [4].

Acids cause coagulation necrosis of the tissues with thrombus formation in the microvasculature of the lesion. Superficial burns occur after only 5 sec of contact and full-thickness burns after 30 sec [1].

Skin contact with nitric acid leads to specific yellow-to- brown-stained wounds by binding with complex proteins (xanthoproteic reaction) and forming a yellow substance called xanthoproteic acid [2, 5], with a possible accumulation of eschar and demarcation compared with thermal burns [6].

There was no specific information about the emergency management or therapies for nitric acid burn traumata in the medical databases [6]. However, in all chemical injuries, the primary concern is the removal of the offending agent because the longer the offending agent is in contact with the skin, the severe the injury becomes. In some situations, an antidote may be given to counteract the offending chemical agent, if it is available, and whatever the agent may be, the initial treatment consists of the removal of saturated clothing, brushing of the skin if the agent is a powder, and irrigation with copious amounts of water [1].

The management can be very different from one practitioner to another knowing that there are no specific guidelines [6]. Therefore, we propose that the patient presenting this kind of an injury should benefit first of all from an intensive lavage (irrigation), which should be continuous from the time of discovery of the injury until emergency evaluation in the hospital, or until definitive treatment is begun, or until the patient experiences a decrease in the pain. Then, we applied a silver sulphadiazine cream and an oily bandage on the lesions [1].

In 2010, Kolios et al. conducted a study by including a total of 24 patients injured with nitric acid [6]. They were treated with an intensive lavage of the injured areas, and then they received a therapy with silver sulphadiazine [6]. Some other treatments were proposed as the external cream (Lavasept gel), foam bandages (Mepilex) in case of chemical wounds (up to IIa° depth), or even an occlusive antiseptic moist bandage in combination with enzymatic substances for deeper wounds (IIb°-III°) (octenidine with phenoxyethanol and octenisept) [6].

The burn wounds are managed after the initial intervention with proper wound care and reconstructive procedure depending on the degree of skin burns [1].

## 4. Conclusion

To conclude, the interest of our publication is to underline the importance and effectiveness (with a concrete example) of the main treatment aims of chemical burn wound management. The early and abundant irrigation with water or sterile isotonic saline solution, the use of panthenol-containing creams and covering with silver sulphadiazine dressing, carefully monitoring wounds, keeping wounds clean and moist, and preventing and managing secondary infection allow the healing.

## Data Availability

The data used to support the findings of this study are available from the corresponding author upon reasonable request.
